# Identification of *Viscum album* L. miRNAs and prediction of their medicinal values

**DOI:** 10.1371/journal.pone.0187776

**Published:** 2017-11-07

**Authors:** Wenyan Xie, Jacob Adolf, Matthias F. Melzig

**Affiliations:** 1 Institut für Pharmazie, Freie Universität Berlin, Berlin, Germany; 2 Technische Hochschule Wildau, Wildau, Germany; Kunming University of Science and Technology, CHINA

## Abstract

MicroRNAs (miRNAs) are a class of approximately 22 nucleotides single-stranded non-coding RNA molecules that play crucial roles in gene expression. It has been reported that the plant miRNAs might enter mammalian bloodstream and have a functional role in human metabolism, indicating that miRNAs might be one of the hidden bioactive ingredients in medicinal plants. *Viscum album* L. (Loranthaceae, European mistletoe) has been widely used for the treatment of cancer and cardiovascular diseases, but its functional compounds have not been well characterized. We considered that miRNAs might be involved in the pharmacological activities of *V*. *album*. High-throughput Illumina sequencing was performed to identify the novel and conserved miRNAs of *V*. *album*. The putative human targets were predicted. In total, 699 conserved miRNAs and 1373 novel miRNAs have been identified from *V*. *album*. Based on the combined use of TargetScan, miRanda, PITA, and RNAhybrid methods, the intersection of 30697 potential human genes have been predicted as putative targets of 29 novel miRNAs, while 14559 putative targets were highly enriched in 33 KEGG pathways. Interestingly, these highly enriched KEGG pathways were associated with some human diseases, especially cancer, cardiovascular diseases and neurological disorders, which might explain the clinical use as well as folk medicine use of mistletoe. However, further experimental validation is necessary to confirm these human targets of mistletoe miRNAs. Additionally, target genes involved in bioactive components synthesis in *V*. *album* were predicted as well. A total of 68 miRNAs were predicted to be involved in terpenoid biosynthesis, while two miRNAs including val-miR152 and miR9738 were predicted to target viscotoxins and lectins, respectively, which increased the knowledge regarding miRNA-based regulation of terpenoid biosynthesis, lectin and viscotoxin expressions in *V*. *album*.

## Introduction

MicroRNAs (miRNAs) are a class of single-stranded non-coding RNA molecules of approximately 22 nucleotides that play crucial roles in gene expression [1]. They generally bind to complimentary sequences in the 3’ untranslated region (UTR) of specific protein-coding genes, inducing mRNA cleavage or translational repression [2]. MiRNAs are highly pleiotropic and a single miRNA can recognize hundreds of mRNA transcripts, allowing them to regulate a diverse range of biological pathways [3,4]. In 2012, Zhang *et al*. reported miRNAs derived from plant-based dietary can function as active signalling molecules to regulate mammalian genes [5]. A recent study demonstrated that a medicinal plant-derived miRNA, MIR2911, can be acquired by mice via GI tract and target influenza A virus and protect the mice against influenza virus infections [6]. These findings provide thrilling clues that miRNAs might act as bioactive constituent mediating the cross-kingdom regulation [7].

*Viscum album* L. (Loranthaceae) commonly known as mistletoe or European mistletoe, is a hemi-parasitic evergreen shrub that grows on a number of host trees including apple, oak, poplar and other trees [8]. Mistletoe has been used medicinally in Europe for centuries. In ancient Greece, Hippocrates (460–377 BC) used the mistletoe to treat disorders of the spleen and complaints associated with menstruation. Around 150 AD, the Roman naturalist Celsus prescribed mistletoe to treat abnormal growths (including possible swellings and cancer). During the middle ages, mistletoe was considered as a golden herb for the treatment of epilepsy. In the 16th century, mistletoe was applied for many conditions including epilepsy, diseases of kidneys and spleen, ulcers, bone fractures and labour-pain. According to the homeopathic *Materia Medica*, mistletoe was applied for “weakness of the heart” and oedema in the 18th century. However, in the late 19th century when the modern medicine rose, these medicinal applications of mistletoe did not gain considerable attention. Until 1907, Gaultier scientifically proved the anti-hypertensive effect of mistletoe extract. In the 1920s, mistletoe was recommended as a possible treatment for cancer. Thereafter, the medicinal use of mistletoe has awakened [9].

Nowadays, *V*. *album* extracts are most frequently used in adjuvant cancer therapy in German-speaking countries [10]. Preparations from *V*. *album* extracts for this purpose are commercially available in Europe, such as Iscador^®^, Eurixor^®^, Helixor^®^ and Abnoba viscum^®^. Three components of mistletoe, namely viscotoxins, lectins and terpenoids, which showed significant immune-system-stimulating activity and cell-killing activity, were suggested to be responsible for its anti-cancer effect [11–13]. In folk medicine, *V*. *album* has been mainly practiced for the treatment of cardiovascular diseases such as hypertension and diabetes [14,15], but its clinical efficacy has not been established [16]. Studies have shown that *V*. *album* extracts possess potent cardioprotective, hypoglycemic, anti-hypertensive and vasodilator effects both *in vitro* and *in vivo* [17–22], nitric oxide pathway, calcium signaling pathway and cholinergic pathway might be involved [18,20,23]. Although various secondary metabolites such as flavonoids, saponins, tannins, alkaloids, phenylpropanoids are present in *V*. *album* [15,20], the bioactive constituents that might be responsible for its cardiovascular protective effects remain to be elucidated [19,24].

In this study, we considered that miRNAs might be involved in the pharmacological activities of *V*. *album*. The conserved and novel miRNAs from *V*. *album* have been identified using Illumina platform technologies. The putative human targets have been predicted using bioinformatics tools, and their potential roles in human biological pathways and diseases have been elucidated. The results indicated that mistletoe miRNAs might possess beneficial effects against some human diseases such as cancer, cardiovascular diseases and neurological disorders, which might explain the medicinal use of mistletoe in ancient time, and provide scientific support for folk medicinal use and clinical use of mistletoe in modern medicine.

Furthermore, to promote understanding of miRNA-based regulation of bioactive ingredients in *V*. *album*, the genes involved in terpenoids, lectins and viscotoxins biosynthesis in *V*. *album* have been characterized, and their corresponding regulatory miRNAs have been predicted, which might facilitate bioengineering research in the production of mistletoe pharmacologically active components.

## Materials and methods

### Ethics statement

The mistletoe plants were collected from Niefern-Öschelbrunn (Baden-Wuerttemberg) Germany with permission of Birken AG. We confirm that the experiments in this study did not involve any endangered or protected species.

### Plant materials

One-year-old leaves and stems were randomly collected from different individual *V*. *album* grown on *Malus domestica* L. trees in Niefern-Öschelbrunn (Baden-Wuerttemberg) Germany in November 2015. The mistletoe plants were snapped in liquid nitrogen and stored at -80°C until use.

### RNA isolation, library construction and high-throughput sequencing

Total RNA was extracted from plant leaves and stems using RNA isolation reagent (amsbio, USA) according to the manufacturer’s protocol. The RNA samples with high purity (OD260/280 between 1.8 and 2.2) and high integrity (RNA integrity number of 6.5 or higher) were used to construct the sRNA library. The mRNA and small RNA library preparations and sequencing were performed by BGI (Beijing Genomics Institute, Shenzhen, China). For mRNA library construction, mRNA in the sample was enriched and fragmented. The RNA fragments were served as templates for cDNA synthesis. The cDNA fragments were ligated with sequencing adapters, and amplified by PCR to construct the cDNA library for paired-end sequencing. Small RNAs (18 to 30 nt) were gel purified and ligated to the 3’ and 5’ adaptor. The ligated products were used for cDNA synthesis, followed by acrylamide gel purification and PCR amplification to generate small RNA library. The Agilent 2100 Bioanalyzer (Agilent, USA) was used for quantification and qualification of the sample library. Finally, the library was sequenced using Illumina HiSeq 4000 sequencing platform (Illumina Inc., San Diego, CA, USA).

### Sequence data analysis

The raw reads obtained from Illumina sequencing were processed by trimming low-quality reads, reads with 5’ adapter contaminants, reads without 3’ adapters, reads without an insert fragment, reads containing poly A, and reads shorter than 18 nt. Other RNAs (rRNA, tRNA, snRNA and snoRNA) were removed by blasting against the GenBank database (http://blast.ncbi.nlm.nih.gov) and the Rfram database (http://rfam.xfam.org/). The remaining clean reads were used to detect conserved and novel miRNAs.

The reads obtained by RNA-seq sequencing were filtered by adaptor sequences, duplication sequences, and low quantity reads. De novo transcriptome assembly was performed by Trinity [25]. The Trinity program first assembles reads of a certain length that overlap to form longer fragments without gaps called contigs. These contigs were further processed for sequence clusters using the sequence clustering software TGICL [26] to obtain unigenes that could no longer be extended on either end. The sequence dataset generated in this study is available at the sequence read archive (SRA) of National Center for Biotechnology Information (NCBI) under the accession numbers of SUB2752327 and SUB2754679.

### Identification of the conserved and putative novel miRNAs

The clean data were used in a BLAST search against known plant miRNAs in the miRBase 21.0, and matched sequences were considered as conserved *V*. *album* miRNAs. The small RNAs that were unaligned to any databases were defined as unannotated sequences.

The novel miRNAs were identified by mapping unannotated sequences to the *V*. *album* transcriptome using Mireap software (http://sourceforge.net/projects/mireap/). The parameters setting for the identification of novel miRNA were: 1) the sequences used to predict novel miRNAs were from the unannotated sequences that were matched to the transcriptome of *V*. *album*; 2) The sequences and their structures satisfy the criteria of forming hairpin miRNAs, and that the mature miRNAs were present in one arm of the hairpin precursors; 3) Hairpin precursors did not contain large internal loops or bulges; 4) Secondary structures of hairpins had free energy of hybridization ≤−18kcal/mol; and 5) The number of mature miRNAs with predicted hairpins were ≥ 5 in the alignment result [27]. The novel miRNAs were named using a “miR” prefix to denote miRNAs, a three-letter prefix to denote the species (e.g. “val” representing *V*. *album*) and a unique sequential number [1].

### Plant targets prediction for both conserved and novel miRNAs

The *V*. *album* conserved and novel miRNA candidates were searched against the *V*. *album* transcriptome database using psRobot (http://omicslab.genetics.ac.cn/psRobot/) and TargetFinder (http://targetfinder.org/) with default parameters to identify potential miRNA target genes. The target candidates were searched against protein database Nr using BLASTX with E-values less than e-5 to predict their possible functions. To classify the function distribution of these potential targets, Gene Ontology (GO) annotation and functional classification were conducted using Blast2GO and WEGO [28,29].

### Human target gene prediction for the novel miRNAs

The novel miRNAs are unique to *V*. *album* and differ from those found in other plant species, and might be responsible for the unique medicinal value of *V*. *album*. These novel miRNAs were therefore used for human targets prediction. In addition, to minimize false positives, the novel miRNAs were further filtered with following conditions: (1) the maximal free energy allowed for the miRNA precursor was -30kcal/mol; (2) the length of precursors were no more than 200nt; (3) the reads for mature miRNAs were at least 20. The human mRNA sequence were download from the UCSC genome browser (http://hgdownload.soe.ucsc.edu/goldenPath/hg19/bigZips/refMrna.fa.gz). Four commonly used animal target prediction algorithms including TargetScan, miRanda, PITA, and RNAhybrid were employed to predict putative human genes, and only those identified by all four softwares were selected for further study [30–33]. The target genes were mapped to the KEGG database to identify significantly enriched metabolic pathways or signal transduction pathways in target genes compared with the whole genome background. A corrected p value <0.05 was set as the threshold.

### Real-time quantitative PCR

Five conserved and five novel miRNAs were randomly selected and validated by stem-loop RT-PCR as previously described by Chen *et al*. [34]. The stem-loop primers for reverse transcription and primers for PCR were listed in S1 Table. First-strand cDNA synthesis was performed using TaqMan MicroRNA Reverse Transcription Kit (Thermo Scientific). The reaction was carried out at 16°C for 30min, at 42°C for 10min, followed by heat-inactivation at 85°C for 5min.

Quantitative real-time PCR was conducted using the PowerUp^™^ SYBR^®^ Green Master Mix (Thermo Scientific) and PikoReal Real-Time PCR System (Thermo Scientific). The reactions were carried out under the following amplification conditions: activation at 50°C for 2min, 95°C for 2min, followed by 40 cycles of denaturation at 95°C for 15s, annealing at 55°C for 15s, and extension then at 72°C for 30s. All reactions were performed in three independent biological samples with three technical repeats. The melting curve was generated to test the specificity of PCR products and avoid the false-positive peaks. No template control and no reverse transcription control were included in all reactions.

## Results

### Analysis of small RNA

**Fig 1:**
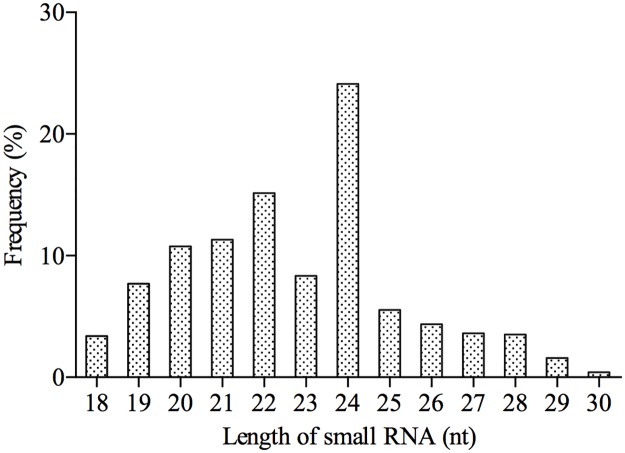
Length distribution of small RNAs from *V*. *album*.

In total, 76357334 raw reads were initially obtained. After data processing, 73552622 clean reads (96.33% of all raw reads) were kept for subsequent analysis. As shown in Fig 1, the clean reads exhibited an uneven length distribution, with the majority (~85%) ranging from 19 to 25nt in length. The most abundant was the small RNAs of 24nt, followed by those of 22, 21 and 20nt. In addition, 342263 (7.75%) unique small RNAs were mapped to the transcriptome data of *V*. *album*. After annotating and removing the non-coding RNAs, including rRNAs, tRNA, snRNAs and snoRNA, 33369 reads remained for the identification of conserved miRNAs, and 4306925 unannotated reads were used for the prediction of novel miRNAs (Table 1).

**Table 1:** Distribution of small RNAs among different categories of *V*. *album*.

Category	Unique small RNAs	Percent (%)	Total small RNAs	Percent (%)
total reads	4415441	100	73552622	100
matched reads*	342263	7.75	44727990	60.81
miRNA	33369	0.76	4245509	5.77
rRNA	58531	1.33	6860606	9.33
snRNA	2030	0.05	92161	0.13
snoRNA	1534	0.03	38977	0.05
tRNA	13052	0.30	1141541	1.55
unannotated	4306925	97.54	61173828	83.17

*The reads that matched to the *V*. *album* transcriptome.

### Identification of conserved miRNAs in *V*. *album*

Evolutionarily conserved miRNAs are present in diverse plant species and play essential roles in plant development and adaptation to adverse environments. The conserved nature of plant miRNAs provides the possibility of finding homolog sequences of miRNAs in different plant species. In this study, 699 conserved miRNAs were identified in *V*. *album* with a total read number of 5511469, of which 44% were detected with more than 100 reads. The most abundant miRNAs were miR166a-3p with 687103 reads, and miR166 with 673128 reads, followed by miR9778 (614703 reads), miR4993 (539038 reads) and miR159a (486726 reads) (S2 Table). Some miRNAs were lowly expressed with abundance of less than ten reads such as miR8004, miR5799 and miR6296 (S2 Table).

### Identification of putative novel miRNAs in *V*. *album*

To identify novel miRNA candidates in *V*. *album*, the unannotated small RNA sequences were matched against the assembled unigene sequences of *V*. *album*. A total of 1373 miRNAs with reads varied from 5 to 11875 were identified as novel miRNA candidates (S3 Table). The length of novel miRNAs ranged from 20 to 23nt, and the precursors ranged from 50 to 372bp in length, with an average of 178bp. The average minimum free energy (MFE) value obtained for these pre-miRNAs was -58.7kcal/mol, which is comparable with the MEF values of precursors for trifoliate orange (*Citrus trifoliate* L. Raf.) (-52.41kcal/mol) [35], *Arabidopsis thaliana* L. (-57kcal/mol) [36], and *Ginkgo biloba var*. *epiphylla* Mak (-46.0kcal/mol) [37]. The first nucleotide bias of these candidate miRNAs was common 5' terminal uridine (U) nucleotide, which is a typical feature of miRNAs [36,38]. The most abundant novel miRNA candidate was val-miR218 with 11875 reads in *V*. *album*, followed by val-miR11 and val-miR1338. Only 4.9% of novel miRNAs were counted more than 20 reads. Although the expression levels of novel miRNA candidates were much lower than the conserved miRNAs, the species-specific functions they played should not be ignored.

### Experimental validation of conserved and novel miRNAs in *V*. *album*

**Fig 2:**
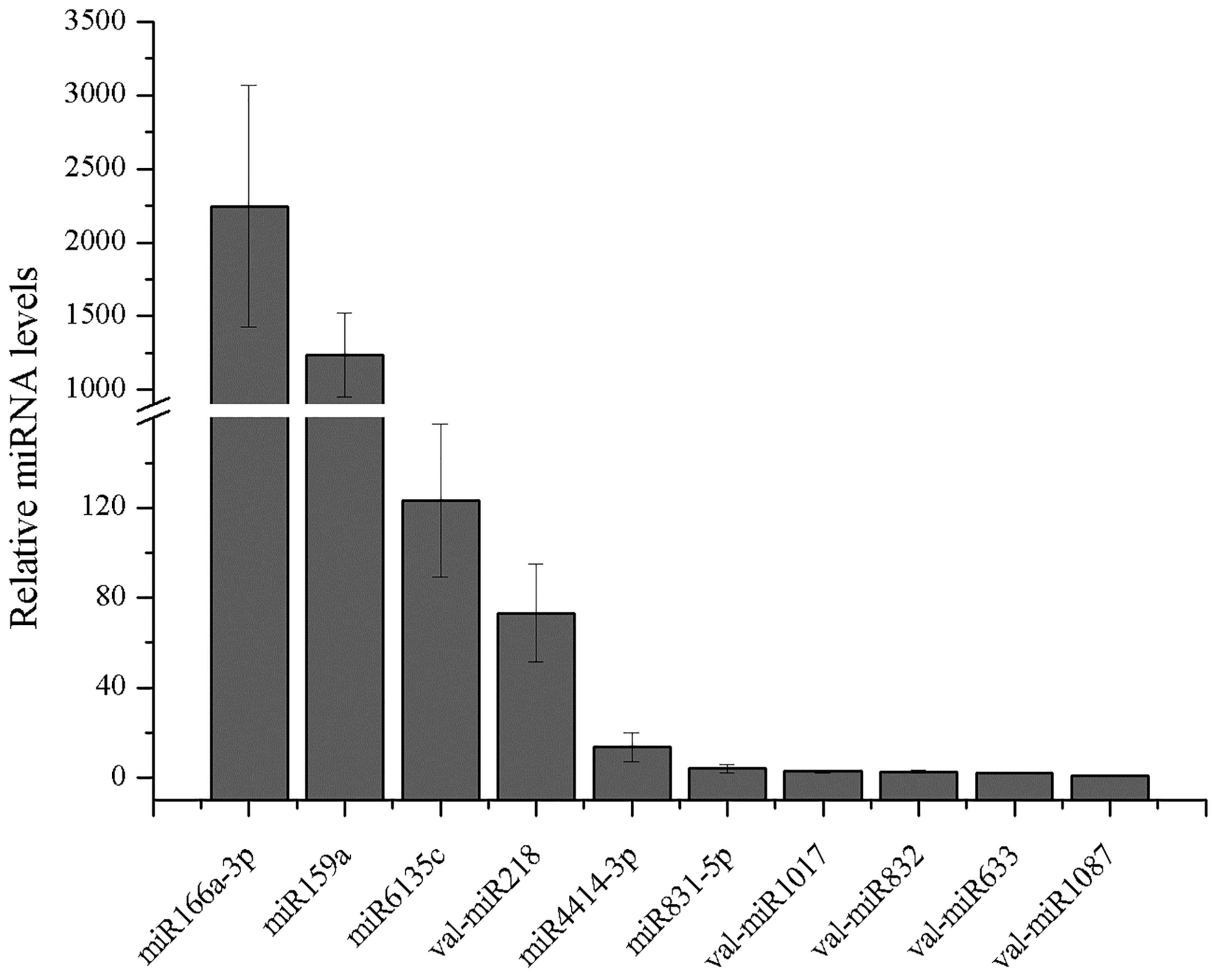
RT-qPCR analysis of miRNAs in *V*. *album*. The expression level of val-miR1087 was set as control and taken as 1, and the expression levels of other miRNAs were quantified relative to it. The values represent the mean and standard deviation of three independent experiments.

Stem-loop RT-qPCR was employed to validate the gene expression data from Illumina sequencing. As illustrated in Fig 2, miR166a-3p was the most abundant miRNA among tested miRNAs, followed by miR159a, miR6135c, val-miR218, miR4414-3p, miR831-5p, val-miR1017, val-miR832, val-miR633 and val-miR1087, respectively. The results from sequencing showed that miR166a-3p, miR159a, miR6135c, val-miR218, miR4414-3p, miR831-5p, val-miR1017, val-miR832, val-miR633 and val-miR1087 with reads of 687103, 486726, 20440, 11875, 5752, 1099, 852, 736, 562 and 285, respectively (S2 and S3 Tables). The expression trend of tested miRNAs was consistent with the Illumina sequencing results, indicating that the gene expression data of miRNAs by sequencing technique was reliable.

### Bioinformatics prediction of *V*. *album* targets for miRNAs

Based on the *V*. *album* transcriptome, a total of 16188 and 17078 target genes were identified for 593 conserved miRNAs and 1373 novel miRNAs, respectively (S4 Table). To evaluate the putative functions, the targets were mapped to Nr database. Many of putative targets were annotated as transcription factors that play important roles in plant growth and development, such as TATA-binding protein (TBP)-associated factor 4 as a potential target of miR5246, miR838-3p, val-miR314 and val-miR1299; transcription initiation factor TFIID as a target of miR838-3p; basic leucine zipper (bZIP) transcription factors predicted to be targeted by miR5380c, val-miR1128, val-miR 885, val-miR273 and val-miR331; MADS-box protein might be targeted by miR8130-5p, miR396e-3p, miR8168, miR477g, miR5293, miR5371-5p and val-miR287.

Besides, some targets encoded proteins involved in stress responses, for example, heat shock protein 70 as target of miR6425a-3p; WRKY transcription factor as target of miR5380c, miR5298b and val-miR799; SNF7 family protein as target of miR477g, miR838-3p and val-miR953; E3 ubiquitin-protein ligase COP1 as target of val-miR132. Other predicted targets encode proteins associated with pollen tube development (val-miR284 for mitochondrial Rho GTPase, val-miR111 for gamma-aminobutyrate transaminase), secondary metabolites synthesis (miR172e-3p and val-miR632 for phenylalanine ammonia-lyase, miR5491, val-miR954 and val-miR477 for omega-hydroxypalmitate O-feruloyl transferase) and immune response (miR8136, val-miR360, val-miR260 and val-miR500 for silencing defective 1 family protein), indicating *V*. *album* miRNAs may be involved in a broad range of physiological and pathological functions.

GO analysis assigned these putative targets into three main categories in terms of biology processes, cellular components, and molecular functions (S5 Table). Based on biology processes, these genes were classified into 23 categories, and the most three over-represented GO terms were “cellular process”, “single-organism process” and “metabolic process”. Categories based on cellular component revealed that these genes were related to 17 cellular parts, of which they are mostly related to “cell”, “cell part” and “organelle”. Based on molecular function, the genes were classified into 14 categories, of which they are mostly involved in “binding” and “catalytic activity” and “transporter activity”.

### Biosynthesis of bioactive components in *V*. *album* and their putative regulatory miRNAs

Terpenoids are one of the main components of mistletoe. The lipophilic extract of *V*. *album* that contained oleanolic acid, betulinic acid, ursolic acid and beta-amyrin acetate showed potent anti-tumor effects [8, 39]. To date, the pathways involved in terpenoids biosynthesis as well as the miRNAs that might regulate these pathways in *V*. *album* are still unclear. Terpenoid precursors can be biosynthesized through the mevalonate (MVA) pathway and/or the methylerythritol phosphate (MEP) pathway in different organisms [40,41]. In current study, most enzymes involved in mevalonate pathway including acetyl-CoA C-acetyltransferase (AACT), 3-hydroxy-3-methylglutaryl-CoA (HMG-CoA) synthase, HMG-CoA reductase, mevalonate kinase, phosphomevalonate kinase and diphosphomevalonate decarboxylase have been identified at transcriptome level (S6 Table). Meanwhile, the genes encoding all the enzymes of the methylerythritol phosphate (MEP) pathway were present in *V*. *album*, including deoxyxylulose-5-phosphate (DOXP) synthase, DOXP reductase, 4-diphosphocytidyl methylerythritol (CDP ME) synthase, CDP ME kinase, methylerythritol cyclodiphosphate (MEcPP) synthase, hydroxymethylbutenyl 4-diphosphate (HMBPP) synthase, HMBPP reductase. These findings indicated that both MVA pathway and MEP pathway were involved in terpenoids backbone biosynthesis in *V*. *album*.

The enzymes of MVA pathway including AACT, HMG-CoA synthase, HMG-CoA reductase and diphosphomevalonate decarboxylase were predicted to be targeted by miR5042-3p, val-miR720; miR477g; miR6196, miR395o-3p, val-miR187; and miR5246, respectively. The DOXP reductase in upstream processes of MEP pathways was predicted to be target of miR8673. All of these aforementioned enzymes are involved in the biosynthesis of isopentenyl diphosphate/dimethylallyl diphosphate, the precursors of the all the downstream end terpenoids. Additionally, some enzymes associated with the biosynthesis of sesquiterpenoid, triterpenoid, monoterpenoid and diterpenoid might be targeted by miRNAs. For example, miR3932b-5p; miR6451, miR9748; miR7820, miR8714, miR9748; miR5258, miR2106 might be able to target beta-amyrin synthase, farnesol dehydrogenase, (+)-neomenthol dehydrogenase and ent-kaurene oxidase, respectively, which are responsible for catalyzing the generation of common triterpene beta-Amyrin, sesquiterpenoid farnesol, monoterpenoid neomenthol and diterpene ent-kaurene, respectively.

Mistletoe lectins (MLs) are complex molecules comprising both protein and carbohydrates that are capable of binding to cells and inducing biochemical changes in cells. Three commonly known toxic lectins MLI, MLII, and MLIII are type II ribosome inactivating proteins (RIPs), and have been reported to stimulate immune system and induce apoptosis in tumor cells [11,42,43]. They share a common primary structure homology but differ in molecular mass and carbohydrate specificity [44]. It was speculated that ML I-III might be encoded by the same gene and process differently during post-translational modification [45]. The sequences CL9238.Contig1 and CL9238.Contig2 are highly homolog to lectin I precursor (99.11%) and lectin precursor (92.31%), respectively (Table 2), and might be responsible for the encoding of ML I-III in *V*. *album*. A structurally unrelated chitin-binding mistletoe lectin that consists of 49 amino acids has been characterized recently [46]. It is less toxic than ML I-III [46]. This lectin shows complete identity (100%) with the protein encoded by Unigene23246, indicating Unigene23246 might be the origin of chitin-binding mistletoe lectin. Besides, two other lectins including Mannose/glucose-specific lectin and Curculin-like (mannose-binding) lectin were also identified in mistletoe, which might possess pharmacological effects [47].

**Table 2:** Genes involved in lectin and viscotoxin expressions and their putative regulatory miRNAs.

Gene name	Accession no.	Putative genes	Identity	E value	miRNAs
lectin I precursor	AAR25545.1	CL9238.Contig1	99.11%	0	NA
lectin precursor	AAR25551.1	CL9238.Contig2	92.31%	3.73E-34	NA
Chitin-binding lectin	P81859.1	Unigene23246	100%	1.78E-26	NA
Mannose/glucose-specific lectin family protein	XP_006372325.1	Unigene7638		2.02E-27	NA
			62.77%		
Curculin-like (mannose-binding) lectin family protein	XP_007021734.1	Unigene24650		5.98E-137	miR9748
			54.77%		
thionin precursor	AAB29761.1	CL146.Contig4	96.49%	1.12E-60	NA
Viscotoxin-A3	AAB29759.1	CL146.Contig1	90.99%	2.01E-53	val-miR152
Viscotoxin-B	P08943.2	CL10031.Contig1	85.15%	2.21E-47	NA

NA represents not available.

Viscotoxins are small proteins belonging to plant thionins, exhibiting cell-killing activity and possible immune-stimulating activity [11,13,15]. To date, seven different isoforms of viscotoxins have been characterized (A1, A1, A3, B, B2, C1 and 1PS), and they differ mainly in their sequence of amino acids. The viscotoxin composition of *V*. *album* depends on its host tree [8]. Consistent with a previous study [48], our results confirmed that viscotoxins A3 and viscotoxin B, which is probably encoded by CL146.Contig1 and CL10031.Contig1, were present in *V*. *album* growing on *Malus*.

As for the potential regulatory miRNAs, only miR9748 and val-miR152 were predicted to target Curculin-like (mannose-binding) lectin family protein and viscotoxin, respectively.

### Bioinformatics prediction of human gene targets for *V*. *album* novel miRNAs

Stringent filters were further applied for the novel miRNA candidates that might target human genes. As listed in Table 3, novel miRNA candidates with more than 20 reads, precursors with minimum free energy of less than -30kcal/mol, and precursors with length of no more than 200nt were considered as the most genuine novel miRNA candidates and were later used for human target prediction.

**Table 3:** Potential novel miRNAs from *V*. *album* used for human target prediction.

miRNA	Reads	Sequences	ML(nt)	PL(nt)	MFE (kcal/mol)
val-miR218	11875	GAUGAUCGCCACGUCGGAGGA	21	119	-63.1
val-miR11	1436	CACUGUAGCACUUUUGACAAAG	22	85	-30.2
val-miR1338	1081	CGCAAGGACGUUAAUGAUGAU	21	143	-43.03
val-miR856	1021	UAAUGGUGCUGGUUCAUGAUCA	22	105	-56.41
val-miR718	882	UUUUGUCUUUGUAGCAUGCUU	21	161	-79.2
val-miR1017	852	UCCCACAUCGGACUUGAGGUC	21	189	-48.4
val-miR832	736	UAAGUUCCAGCUCUGACUACC	21	136	-49.9
val-miR457	565	UAGCCGGGUCUUCUUCAACGC	21	182	-74.77
val-miR633	562	UCAAUGAACUGGGUUGUGCCU	21	105	-53.3
val-miR1370	436	UUCAAUAAAGCUGUGGGAAG	20	98	-48.9
val-miR588	435	CGAUCUGAUAAUUCAAGAUUU	21	126	-50.4
val-miR539	366	UAAAGUCGUAGCAGGUGUCGA	21	131	-44.7
val-miR1087	285	GGGGAAUGACAACUGGGGACC	21	179	-97
val-miR944	278	UUUUUCUUGGUUGGCUUGGGU	21	156	-82.66
val-miR1048	267	CGGUGGAACCUGGCAGUGGG	20	64	-51
val-miR262	227	UUAAAUCCCUGGAUUGGUCUC	21	131	-63.1
val-miR765	201	CAGGAUGGAGAAGCAGGGCAC	21	146	-70.8
val-miR1052	181	CAAGACAUUACUGUGGGCUCC	21	117	-36.13
val-miR421	103	UUUCAGUAAGUGUUGUCGAAC	21	119	-30.4
val-miR885	103	UUGAUUUCAGGAAUAAUGCUC	21	104	-43.3
val-miR64	101	AGGGUGGAUGGAUCGGUGAGA	21	85	-43.7
val-miR790	93	UAGCCAAGGAUGACUUGCCUG	21	111	-54.6
val-miR648	84	UGUCUGAUUAGAACUCCACAGU	22	81	-31.6
val-miR333	80	AUGAUGUCGGAGUAUUUGGCA	21	85	-30
val-miR503	76	UCAUCAAUAUGUUGGUCUGA	21	149	-80
val-miR552	48	CAUUGGAUCUGUAAUUGGACC	21	150	-54
val-miR1306	40	CAAUGGAUGGCCGUCACGUCG	21	108	-68.61
val-miR269	36	GACUACGAUCGGAGGACCCGGG	22	142	-51.1
val-miR1328	33	CGAGAAUGUAGGUCAAGGGCAC	22	127	-39.8
val-miR1127	32	CCCACACUUGAAUGUCGGUG	20	170	-67.8
val-miR1110	28	CGGCGAGGCGAUCGGAGCUCCG	22	91	-46.8
val-miR855	27	GAACUACGUGGACUUUGAUCCU	22	129	-36.3
val-miR1086	26	UGCCCCGGGAUCGUCGAUGCC	21	141	-57.9
val-miR6	26	AUAUGUGAUGUCAAAUGGACC	21	148	-46.41
val-miR954	26	UCUUGGACUGCCGGCGAGCCU	21	130	-56.6
val-miR198	24	UGACGAUUGGGGACCAAAACU	21	148	-69.39
val-miR560	24	UCUCAGCAACUCUGAAUCUGC	21	180	-56.4
val-miR82	24	UGAUUGCUCUCACUCUGGCCU	21	178	-58.5
val-miR1342	23	ACCUUGACCCGUAGGGCUGCA	21	104	-33.39
val-miR92	22	AAGAUCAUGAUCCAAUAGGCCU	22	59	-36.5
val-miR550	21	UCUUUGGGUAUUAGGGGGGAC	21	117	-59.3
val-miR615	21	UUUCUGUCGGCAGCUCGAGGA	21	125	-42.5
val-miR163	20	GAUCGAGGAGUAAGUUAACU	20	197	-84.8
val-miR834	20	CGGCACUCGCGUCUCUGGCC	20	102	-34.6

ML: mature miRNA length; PL, precursor length; MFE, minimum free energy.

As listed in Table 4, the putative human targets vary by different programs. TargetScan, miRanda, PITA, and RNAhybrid predicted thousands of potential human genes for every novel miRNA, while PITA predicted potential targets for 29 miRNAs, which was probably caused by different algorithms and parameters used in different miRNA target prediction programs. It has been suggested that not a single program was consistently superior than the others among current miRNA target prediction programs [49]. Based on the combination of the methods, the intersection of 30697 potential genes with 59266 miRNA-target pairs were used in subsequent bioinformatics analysis (S7 Table).

**Table 4:** Statistics of miRNA target predictions.

miRNA target prediction program	miRNA number	Target genes	miRNA-target pairs
Targetscan	44	46555	581567
miRanda	44	36284	95353
PITA	29	48154	1009051
RNAhybrid	44	48354	1524020
Intersection	29	30697	59266

Molecular components often interact with each other in a complex reaction network to perform certain biological functions. Pathway enrichment analysis identifies significantly enriched metabolic pathways and signal transduction pathways in proposed targets comparing with the whole genome background, which helps further elucidating genes biological functions. The predicted targets were mapped to the KEGG database and categorized into 305 pathways of which 33 signaling pathways were significantly enriched (Table 5). Notably, these highly enriched KEGG pathways are associated with some human diseases, especially cancer, cardiovascular diseases and neurological disorders.

**Table 5:** Highly enriched KEGG pathways for putative human targets.

Pathway terms	Number of target genes	Rich factor	Qvalue	Pathway ID
MAPK signaling pathway	793	0.795	7.71E-10	ko04010
Transcriptional misregulation in cancer	1866	0.755	1.89E-08	ko05202
Calcium signaling pathway	495	0.810	2.41E-08	ko04020
VEGF signaling pathway	271	0.850	2.43E-08	ko04370
ECM-receptor interaction	365	0.824	6.91E-08	ko04512
Purine metabolism	1684	0.750	8.92E-07	ko00230
Glutamatergic synapse	312	0.823	9.30E-07	ko04724
Focal adhesion	692	0.773	1.37E-05	ko04510
Neurotrophin signaling pathway	421	0.794	1.52E-05	ko04722
Pyrimidine metabolism	1488	0.746	4.01E-05	ko00240
Measles	309	0.794	4.00E-04	ko05162
Morphine addiction	219	0.808	7.92E-04	ko05032
GnRH signaling pathway	273	0.796	7.92E-04	ko04912
Phosphatidylinositol signaling system	229	0.801	1.49E-03	ko04070
Arginine and proline metabolism	404	0.774	1.57E-03	ko00330
Type II diabetes mellitus	188	0.810	1.57E-03	ko04930
Axon guidance	440	0.769	1.91E-03	ko04360
GABAergic synapse	214	0.799	2.69E-03	ko04727
Pathways in cancer	874	0.746	3.37E-03	ko05200
Herpes simplex infection	407	0.768	3.71E-03	ko05168
Retrograde endocannabinoid signaling	230	0.785	9.05E-03	ko04723
Glycosaminoglycan biosynthesis—chondroitin sulfate	40	0.909	1.20E-02	ko00532
Alanine, aspartate and glutamate metabolism	93	0.830	1.47E-02	ko00250
Influenza A	448	0.755	1.69E-02	ko05164
Amphetamine addiction	246	0.776	1.69E-02	ko05031
Leishmaniasis	174	0.791	1.70E-02	ko05140
Fc epsilon RI signaling pathway	211	0.781	1.76E-02	ko04664
B cell receptor signaling pathway	252	0.771	2.64E-02	ko04662
Pancreatic cancer	195	0.780	2.83E-02	ko05212
beta-Alanine metabolism	226	0.771	3.70E-02	ko00410
Nicotine addiction	86	0.819	3.73E-02	ko05033
Toxoplasmosis	267	0.763	4.63E-02	ko05145
Non-small cell lung cancer	147	0.786	4.63E-02	ko05223

Rich factor represents the ratio of the number of predicted genes and the number of all genes in the pathway.

Five significant enriched signaling pathways are highly related to cancer including transcriptional misregulation in cancer (ko05202), vascular endothelial growth factor (VEGF) signaling pathway (ko04370), pathways in cancer (ko05200), pancreatic cancer (ko05212) and non-small cell lung cancer (ko05223). Tumorigenesis is a multistep process involving a series of genetic alterations [50]. Transcription factors play instrumental functions in driving these gene expressions, whereas misregulation of transcription factors can cause the acquisition of tumor-related properties [51]. For example, runt related transcription factor 1 (RUNX1) and lysine methyltransferase 2A (MLL1) are essential for chromosomal translocations in acute myeloid leukemia [52,53], which were predicted as targets of val-miR1086, val-miR765, val-miR615; and val-miR834 val-miR765, val-miR550, val-miR1127, val-miR954, val-miR1086, val-miR421, respectively. It is well known that the tumor protein p53 is a major tumor suppressor, and the mutation of p53 can provoke tumor imitation [54]. The miRNAs such as val-miR1086 and val-miR1127 were predicted to regulate the expression level of p53. By targeting these genes associated with transcriptional misregulation in cancer, these novel miRNA candidates may prevent cancer initiation and progression. Angiogenesis induction is one of the major hallmarks of cancer. It is generally accepted that VEGF is a major driver of the angiogenic process in physiological and pathological processes. VEGF and its receptors are often found overexpressed in tumors [55,56]. Suppression of the essential molecules in VEGF signaling pathway, such as phospholipase C gamma 1 (PLCG1) possibly targeted by val-miR615 and val-miR1086, tyrosine-protein kinase Src possibly targeted by val-miR834 and val-miR1086, may block the angiogenic activity of tumor tissue, resulting in tumor vascular regression and anti-tumor effects.

Diabetes mellitus is one of the most prevalent metabolic disorders. It is characterized by hyperglycemia, and long-term hyperglycemia may lead to systemic complications, such as macrovascular diseases, coronary artery disease, peripheral arterial disease and stroke [57]. Insulin receptor (INSR) dysregulation is a well-established defect in type II diabetes mellitus (ko04930). Mitogen-activated protein kinase 1(ERK) and inhibitor of nuclear factor kappa B kinase (IKK) are serine kinases that can directly inactivate insulin receptor substrate (IRS) through serine phosphorylation, and impair insulin sensitivity [58,59]. The putative inhibition of ERK and IKK by val-miR615 and val-miR834 might restore the impaired INSR singling. Calcium is a critical mediator of excitation–contraction coupling in cardiac cells, and cellular calcium signaling dysfunction is central to the pathophysiology of a wide range of cardiac diseases [60]. Based on computational analysis, a range of key molecules in calcium signaling pathway (ko04020) were targeted by mistletoe miRNAs, for example, calcium voltage-gated channels (CaV1, CaV2 and CaV3) were mutual putative targets of val-miR1086 and val-miR765, calcineurin (CaN) was predicted to be target by val-miR765 and val-miR1110. Through regulation of these potential therapeutic targets [61,62], mistletoe miRNAs might be responsible for its cardiovascular protective effect.

It is noteworthy that the putative human genes targeted by mistletoe miRNAs were also involved in several pathways associated with the nervous system, such as neurotrophin signaling pathway (ko04722), morphine addiction (ko05032), glutamatergic synapse (ko04724), GABAergic synapse (ko04727), axon guidance (ko04360), amphetamine addiction (ko05031) and nicotine addiction (ko05033). Mistletoe had been beneficial for the treatment of epilepsy, depression, sleep disorders and labour-pain in middle ages [63], however, since deficiency of scientific evidence, mistletoe preparations are not applied for neurological diseases in modern medicine. Based on our bioinformatics prediction, mistletoe novel miRNAs might target critical neurotransmitter receptors and neurotransmitter transporters, such as gamma-aminobutyric acid type B receptor (GABA_B_) as a putative target of val-miR1086; glutamate metabotropic receptors (mGluRs) as putative targets of val-miR1342, val-miR954, val-miR550, val-miR560, val-miR765, val-miR1086, val-miR550, val-miR1328; dopamine transporter (DAT) as a putative target of val-miR765 and val-miR1110. It is possible that mistletoe miRNAs could influence neurotransmission by affecting transport of neurotransmitters including GABA, glutamic acid and dopamine, which might explain the traditional use of mistletoe to treat epilepsy, insomnia and other neurological disorders. One critical concern regarding treatment of neurological diseases is the blood-brain barrier that represents a problem to any therapy involving systemic delivery of oligonucleotides [64]. Recent publications indicated that exosomes could transfer across blood-brain barrier, serving as an efficient vehicle to deliver miRNAs to the recipient neurocytes [65–67]. Therefore, with the assistance of plant- or animal-derived molecules or nanoparticles such as exosomes, mistletoe miRNAs might reach nervous system and exert its function.

Interestingly, it is recorded that mistletoe has been used by North American Indians to treat measles and dog bites [68], indicating the antiviral potential of mistletoe. Indeed, the predicted targets were involved in several virus and parasitic infections including measles (ko05162), herpes simplex infection (ko05168), influenza A (ko05164), leishmaniasis (ko05140) and toxoplasmosis (ko05145). Specifically, Toll-like receptors that are sentinel receptors of the host innate immune system to detect the presence of microbial infection [69], were predicted to be targeted by val-miR765 and val-miR1328. Furthermore, val-miR1086 and val-miR954 might target TNF receptor (TNFR) and interferon gamma receptor (IFNGR), and thus prevent overexuberant inflammatory response. Although mistletoe is not used for pathogenic diseases nowadays, the antiviral potential of mistletoe might be recognized.

## Discussion

Endogenous microRNAs (miRNAs) are a class of single-stranded non-coding RNA molecules of approximately 22 nucleotides that play crucial roles in gene expression. In mammals, an estimated 60% of all protein-coding genes may contain miRNA binding sites [3,4]. MiRNA dysregulation is frequently associated with human diseases such as cancer, cardiovascular diseases, central nervous system diseases and metabolic disorders [70,71]. To date, miRNA-based novel therapeutics have been developed for the treatment of human diseases, and several preclinical studies on therapeutic miRNA replacement have been initiated [72,73], indicating miRNA-based therapeutics are coming of age.

Herbal medicine is globally accepted as a valid alternative system of therapy. Though ancient medical treatises have documented a large number of medicinal plants, their bioactive constituents and corresponding interactions with human have not been comprehensively characterized. New plant bioactive molecules are being discovered. In recent years, regulation of human genes by plant miRNAs has attracted great attention. Rice miRNAs were suggested to enter mammalian bloodstream and have a functional role in human metabolism [5]. The MIR2911 from honeysuckle were found to target influenza viruses and protect mice from influenza [6]. Plant derived miR159 significantly suppressed breast cancer cell proliferation by targeting transcription factor 7 (TCF7) [74]. Oral application of a cocktail that consisted of plant-based tumour suppressor miRNAs was able to reduce tumour burden in mice [75]. These studies indicate that miRNAs derived from plants may function as bioactive constituents to regulate human health.

*V*. *album* is a European medicinal plant surrounded by legends and myths. It has been used in folk medicine for the treatment of cancer, cardiovascular disease, and other symptoms. In modern medicine, *V*. *album* has been mainly used as an anti-tumor therapy, which is attributed to the anti-cancer and immune stimulating activities of its bioactive components including viscotoxins, lectins and terpenoids. However, the active ingredients that might be responsible for its cardiovascular protective effects as well as other beneficial applications remain to be clarified.

Here we propose that miRNAs in *V*. *album* might serve as an independent category of active ingredients and provide beneficial effects for human consumers. Since *V*. *album* genome information is limited, we conducted RNA-seq and sRNA-seq to identify and characterize the conserved and possibly novel miRNAs from *V*. *album*. Bioinformatics tools have been applied to understand their possible functions in plant biological processes and potential roles in human gene regulation.

By using high-throughput sequencing technology, a total of 699 conserved miRNAs and 1373 novel miRNAs with a length of 21–24 nt have been identified from *V*. *album*. The reliability of the sequencing data was confirmed by qRT-PCR. In *V*. *album*, these miRNAs were involved in various biological processes including plant growth, development, signal transduction and stress responses. Transcription factors are involved in important plant developmental processes. The MADS-box transcription factors are crucial for floral development [76], and might be controlled by several miRNAs such as miR8130-5p, miR396e-3p and miR8168. WRKY transcription factors are involved in various plant processes, especially in coping with diverse biotic and abiotic stresses [77], and were predicted to be targeted by miR5380c, miR5298b and val-miR799. One miRNA could target several transcription factors, such as miR838-3p was predicted to target TBP-associated factor and TFIID; miR5380c might target bZIP transcription factors and WRKY transcription factors, indicating their multiple roles in plant processes. Besides, the annotated targets involved in various metabolic processes, stimulus response, catalytic activity, and other biological process were also predicted, suggesting that miRNAs play essential roles in plant growth and development.

Mistletoe lectins and viscotoxins are pharmaceutical proteins present in *V*. *album*, and they have been considered to be mainly responsible for the anti-tumor activity of *V*. *album*. Previous studies have isolated and identified lectins and viscotoxins from mistletoe at protein level [44,46,78,79]. In this study, the expressions of these bioactive components were confirmed at transcriptome level. ML I-III might be translated from CL9238.Contig1 and/or CL9238.Contig2, while Unigene23246 probably encodes chitin-binding mistletoe lectin. However, the miRNAs that might target CL9238.Contig1, CL9238.Contig2 and Unigene23246 were not identified in our study. It has been reported that the amount of MLs in the leaves of *V*. *album* showed maximum in December [80]. It is possible that at this time, their corresponding regulatory miRNAs are too low to be detected. Mannose/glucose-specific lectin and curculin-like (mannose-binding) lectin, which differ from known mistletoe lectins (ML I-III and chitin-binding lectin), have been newly identified from mistletoe. However, the expression and bioactivities of these two newly identified mistletoe lectins need to be further validated.

For viscotoxins, CL146.Contig1, which is highly homologous to Viscotoxin-A3 (90.99%), was predicted to be target of val-miR152. However, no miRNAs were identified to target CL10031.Contig1 and CL146.Contig4, which were annotated as Viscotoxin-B and thionin precursor, respectively. Except through post-transcriptional regulation by miRNAs, the expressions of viscotoxins might as well be controlled by transcriptional regulation.

The pharmacological properties of *V*. *album* also attributed to the presence of triterpene acids, especially oleanolic acid, betulinic acid and ursolic acid, which have been reported to enhance the toxicity of mistletoe lectins in tumor cells [39,42]. The biosynthesis of terpenoids in *V*. *album* has not yet been elucidated. Our study identified majority of genes encoding enzymes that involved in both MVA pathway and MEP pathway, indicating terpenoids biosynthesis in *V*. *album* was via both pathways. The compounds isopentenyl pyrophosphate (IPP) and dimethylallyl pyrophosphate (DMAPP), produced in upstream pathway, are the common precursors for all the downstream end terpenoids. The miRNAs such as miR5042-3p, miR477g and miR6196 that predicted to target the upstream enzymes, might be involved in the regulation of IPP and DMAPP levels. Some putative targets of miRNAs were downstream enzymes in mono-, sesqui, di-, and triterpenoid biosynthetic pathways, such as beta-amyrin synthase, farnesol dehydrogenase, neomenthol dehydrogenase and ent-kaurene oxidase might be targeted by miR3932b-5p; miR6451, miR9748; miR7820, miR8714, miR9748; miR5258, miR2106, respectively.

Bioinformatics predictions have been employed to identify the potential human targets of plant miRNAs. Seven miRNAs from medicinal plant *Moringa oleifera* L., have been predicted to involve in cell cycle, apoptosis and metabolic regulation in humans [81]. Approximately 50 human target genes associated with energy metabolism, lipoprotein metabolism, and other biological process have identified as the target genes of a rice miRNA (MIR168a) [5]. In this study, bioinformatics tools were applied to identify the putative human target genes of 44 *V*. *album* specific miRNAs. A huge number of 30697 putative genes were predicted and then mapped to the KEGG database to find their roles in human metabolism and human diseases. A total of 14995 putative targets were highly enriched in 33 KEGG pathways. Among them, five pathways were highly related with cancer including transcriptional misregulation in cancer (ko05202), VEGF signaling pathway (ko04370), pathways in cancer (ko05200), pancreatic cancer (ko05212), non-small cell lung cancer (ko05223), while Type II diabetes mellitus (ko04930) and calcium signaling pathway (ko04020) were associated with cardiovascular and metabolism diseases. By targeting essential molecules involved in these pathways, *V*. *album* specific miRNAs might possess pharmaceutical effects against cancer, cardiovascular and metabolism diseases, which might provide scientific support for the folk and clinic use of mistletoe.

The use of mistletoe for the treatment of neurological disorders and infections has been recorded in ancient times. However, since there is no scientific evidence explaining its effects, mistletoe is not used for these purposes in modern medicine. Interestingly, bioinformatics predictions showed that some of the putative targets relate to several neurological pathways, including neurotrophin signaling pathway (ko04722), morphine addiction (ko05032), glutamatergic synapse (ko04724), GABAergic synapse (ko04727), axon guidance (ko04360), amphetamine addiction (ko05031) and nicotine addiction (ko0533). Some infections related pathways such as measles (ko05162), herpes simplex infection (ko05168), influenza A (ko05164), leishmaniasis (ko05140) and toxoplasmosis (ko05145) were also highly enriched. These findings might provide an explanation for the traditional medicine use of mistletoe in middle ages, and inspire the modern medicine use of mistletoe.

Experimental validation of predicted plant miRNA-human mRNA interaction is necessary in upcoming investigations. However, a series of questions remain to be answered. Would herbal miRNAs be stable during herbal preparation and human digestion process? Would herbal miRNAs be selectively absorbed by the human gastrointestinal tract? How would plant miRNAs be recognized by human cells? How would plant miRNAs be loaded into mammalian RNA Induced Silencing Complex (RISC), in which the miRNAs exert their function together with Argonaute proteins? Our study implied that medicinal plant specific miRNAs might contribute to their corresponding pharmaceutical effects, and our next step would be to focus on the detection of herbal miRNAs in various herbal preparations, evaluation of the capability of herbal miRNAs to transfer intestinal barriers, and investigation of their intracellular fate in human cells.

In summary, this study comprehensively identified the miRNAs from medicinal plant *V*. *album*, and characterized the genes and their potential regulatory miRNAs for the synthesis of bioactive components such as viscotoxins, lectins and terpenoids, helping to develop a deeper understanding of biosynthesis of active ingredients in mistletoe. Computational predictions indicated the anti-tumor potential, cardiovascular protective and neurological protective effects of *V*. *album* specific miRNAs, and initiated further investigation to elucidate the regulatory function of plant miRNAs in human health and diseases.

## Supporting information

S1 Table Primers for qRT-PCT. (XLSX)

S2 Table Conserved miRNAs identified in *V*. *album*. (XLSX)

S3 Table Novel miRNA candidates from *V*. *album*. (XLSX)

S4 Table Predicted *V*. *album* targets for conserved and novel miRNAs. (XLSX)

S5 Table GO analysis of target genes for miRNAs in *V*. *album*. (XLSX)

S6 Table Target genes for miRNAs involved in terpenoids biosynthesis. (XLSX)

S7 Table Putative human targets of *V*. *album* novel miRNAs. (XLSX)

## References

[1] Ambros V, Bartel B, Bartel DP, Burge CB, Carrington JC, Chen X, et al A uniform system for microRNA annotation. RNA 2003; 9: 277–279 doi: 10.1261/rna.2183803 12592000 10.1261/rna.2183803 PMC1370393

[2] Ling H, Fabbri M, Calin GA. MicroRNAs and other non-coding RNAs as targets for anticancer drug development. Nat Rev Drug Discov 2013; 12: 847–865 doi: 10.1038/nrd4140 24172333 10.1038/nrd4140 PMC4548803

[3] Friedman RC, Farh KK-H, Burge CB, Bartel DP. Most mammalian mRNAs are conserved targets of microRNAs. Genome Res 2009; 19: 92–105 doi: 10.1101/gr.082701.108 18955434 10.1101/gr.082701.108 PMC2612969

[4] Ranganathan K, Sivasankar V. MicroRNAs—Biology and clinical applications. J Oral Maxillofac Pathol 2014; 18: 229–234 doi: 10.4103/0973-029X.140762 25328304 10.4103/0973-029X.140762 PMC4196292

[5] Zhang L, Hou D, Chen X, Li D, Zhu L, Zhang Y, et al Exogenous plant MIR168a specifically targets mammalian LDLRAP1: evidence of cross-kingdom regulation by microRNA. Cell Res 2012; 22: 273–274 10.1038/cr.2011.158 PMC3351925 21931358

[6] Zhou Z, Li X, Liu J, Dong L, Chen Q, Liu J, et al Honeysuckle-encoded atypical microRNA2911 directly targets influenza A viruses. Cell Res 2014; 25: 1–11 25287280 10.1038/cr.2014.130 PMC4650580

[7] Xie W, Weng A, Melzig M. MicroRNAs as New Bioactive Components in Medicinal Plants. Planta Med 2016; 82: 1153–1162 doi: 10.1055/s-0042-108450 27272400 10.1055/s-0042-108450

[8] Singh BN, Saha C, Galun D, Upreti DK, Bayry J, Kaveri SV. European Viscum album: a potent phytotherapeutic agent with multifarious phytochemicals, pharmacological properties and clinical evidence. R Soc Chem 2016; 6: 23837–23857

[9] Buessing A. Mistletoe: The Genus Viscum. Harwood academic publishers; 2000

[10] Horneber MA, Bueschel G, Huber R, Linde K, Rostock M. Mistletoe therapy in oncology. Cochrane database Syst Rev 2008; CD003297 10.1002/14651858.CD003297.pub2 PMC7144832 18425885

[11] Twardziok M, Kleinsimon S, Rolff J, Jäger S, Eggert A, Seifert G, et al Multiple Active Compounds from Viscum album L. Synergistically Converge to Promote Apoptosis in Ewing Sarcoma. PLoS One 2016; 11: e0159749 doi: 10.1371/journal.pone.0159749 27589063 10.1371/journal.pone.0159749 PMC5010293

[12] Podlech O, Harter PN, Mittelbronn M, Pöschel S, Naumann U. Fermented mistletoe extract as a multimodal antitumoral agent in gliomas. Evid Based Complement Alternat Med 2012; 2012: 501796 doi: 10.1155/2012/501796 23133496 10.1155/2012/501796 PMC3485514

[13] Tabiasco J, Pont F, Fournié J-J, Vercellone A. Mistletoe viscotoxins increase natural killer cell-mediated cytotoxicity. Eur J Biochem 2002; 269: 2591–2600 12027898 10.1046/j.1432-1033.2002.02932.x

[14] Eddouks M, Maghrani M, Lemhadri A, Ouahidi M-L, Jouad H. Ethnopharmacological survey of medicinal plants used for the treatment of diabetes mellitus, hypertension and cardiac diseases in the south-east region of Morocco (Tafilalet). J Ethnopharmacol 2002; 82: 97–103 12241983 10.1016/s0378-8741(02)00164-2

[15] Nazaruk J, Orlikowski P. Phytochemical profile and therapeutic potential of Viscum album L. Nat Prod Res 2016; 30: 373–385 doi: 10.1080/14786419.2015.1022776 25813519 10.1080/14786419.2015.1022776

[16] PDQ Integrative, Alternative, and Complementary Therapies Editorial Board. Mistletoe Extracts (PDQ^®^): Health Professional Version In: PDQ Cancer Information Summaries. Bethesda (MD): National Cancer Institute (US); 2002

[17] Orhan DD, Aslan M, Sendogdu N, Ergun F, Yesilada E. Evaluation of the hypoglycemic effect and antioxidant activity of three Viscum album subspecies (European mistletoe) in streptozotocin-diabetic rats. J Ethnopharmacol 2005; 98: 95–102 doi: 10.1016/j.jep.2004.12.033 15763369 10.1016/j.jep.2004.12.033

[18] Karagöz A, Kesici S, Vural A, Usta M, Tezcan B, Semerci T, et al Cardioprotective effects of Viscum album L. ssp. album (Loranthaceae) on isoproterenol-induced heart failure via regulation of the nitric oxide pathway in rats. Anatol J Cardiol 2016; 16: 923–930 doi: 10.14744/AnatolJCardiol.2016.6780 27443473 10.14744/AnatolJCardiol.2016.6780 PMC5324911

[19] Deliorman D, Caliş I, Ergun F, Doğan BS, Buharalioğlu CK, Kanzik I. Studies on the vascular effects of the fractions and phenolic compounds isolated from Viscum album ssp. album. J Ethnopharmacol. 2000; 72: 323–329. 10967490 10.1016/s0378-8741(00)00251-8

[20] Khan T, Ali S, Qayyum R, Hussain I, Wahid F, Shah AJ. Intestinal and vascular smooth muscle relaxant effect of Viscum album explains its medicinal use in hyperactive gut disorders and hypertension. BMC Complement Altern Med 2016; 16: 251 doi: 10.1186/s12906-016-1229-3 27465545 10.1186/s12906-016-1229-3 PMC4963958

[21] Tenorio FA, del Valle L, González A, Pastelín G. Vasodilator activity of the aqueous extract of Viscum album. Fitoterapia 2005; 76: 204–209 doi: 10.1016/j.fitote.2004.12.013 15752631 10.1016/j.fitote.2004.12.013

[22] Radenkovic M, Ivetic V, Popovic M, Brankovic S, Gvozdenovic L. Effects of Mistletoe (Viscum Album L., Loranthaceae) Extracts on Arterial Blood Pressure in Rats Treated with Atropine Sulfate and Hexocycline. Clin Exp Hypertens 2009; 31: 11–19 doi: 10.1080/10641960802409820 19172455 10.1080/10641960802409820

[23] Radenkovic M, Ivetic V, Popovic M, Mimica-Dukic N, Veljkovic S. Neurophysiological effects of mistletoe (Viscum album L.) on isolated rat intestines. Phyther Res 2006; 20: 374–377 10.1002/ptr.1865 16619366

[24] Choudhary MI, Maher S, Begum A, Abbaskhan A, Ali S, Khan A, et al Characterization and antiglycation activity of phenolic constituents from Viscum album (European Mistletoe). Chem Pharm Bull (Tokyo) 2010; 58: 980–982 20606351 10.1248/cpb.58.980

[25] Grabherr MG, Haas BJ, Yassour M, Levin JZ, Thompson DA, Amit I, et al Full-length transcriptome assembly from RNA-Seq data without a reference genome. Nat Biotechnol 2011; 29: 644–652 doi: 10.1038/nbt.1883 21572440 10.1038/nbt.1883 PMC3571712

[26] Pertea G, Huang X, Liang F, Antonescu V, Sultana R, Karamycheva S, et al TIGR Gene Indices clustering tools (TGICL): a software system for fast clustering of large EST datasets. Bioinformatics 2003; 19: 651–652 12651724 10.1093/bioinformatics/btg034

[27] Meyers BC, Axtell MJ, Bartel B, Bartel DP, Baulcombe D, Bowman JL, et al Criteria for Annotation of Plant MicroRNAs. PLANT CELL ONLINE 2008; 20: 3186–3190 10.1105/tpc.108.064311 PMC2630443 19074682

[28] Conesa A, Gotz S, Garcia-Gomez JM, Terol J, Talon M, Robles M. Blast2GO: a universal tool for annotation, visualization and analysis in functional genomics research. Bioinformatics 2005; 21: 3674–3676 doi: 10.1093/bioinformatics/bti610 16081474 10.1093/bioinformatics/bti610

[29] Ye J, Fang L, Zheng H, Zhang Y, Chen J, Zhang Z, et al WEGO: a web tool for plotting GO annotations. Nucleic Acids Res 2006; 34: W293–7 doi: 10.1093/nar/gkl031 16845012 10.1093/nar/gkl031 PMC1538768

[30] Krüger J, Rehmsmeier M. RNAhybrid: microRNA target prediction easy, fast and flexible. Nucleic Acids Res 2006; 34: W451–4 doi: 10.1093/nar/gkl243 16845047 10.1093/nar/gkl243 PMC1538877

[31] Enright AJ, John B, Gaul U, Tuschl T, Sander C, Marks DS. MicroRNA targets in Drosophila. Genome Biol 2003; 5: R1 doi: 10.1186/gb-2003-5-1-r1 14709173 10.1186/gb-2003-5-1-r1 PMC395733

[32] Lewis BP, Shih I, Jones-Rhoades MW, Bartel DP, Burge CB. Prediction of mammalian microRNA targets. Cell 2003; 115: 787–798 14697198 10.1016/s0092-8674(03)01018-3

[33] Cheng C, Bhardwaj N, Gerstein M. The relationship between the evolution of microRNA targets and the length of their UTRs. BMC Genomics 2009; 10: 431 doi: 10.1186/1471-2164-10-431 19751524 10.1186/1471-2164-10-431 PMC2758905

[34] Chen C, Ridzon DA, Broomer AJ, Zhou Z, Lee DH, Nguyen JT, et al Real-time quantification of microRNAs by stem-loop RT-PCR. Nucleic Acids Res 2005; 33: e179 doi: 10.1093/nar/gni178 16314309 10.1093/nar/gni178 PMC1292995

[35] Sun LM, Ai XY, Li WY, Guo WW, Deng XX, Hu CG, et al Identification and Comparative Profiling of miRNAs in an Early Flowering Mutant of Trifoliate Orange and Its Wild Type by Genome-Wide Deep Sequencing. PLoS One 2012; 7: e43760 doi: 10.1371/journal.pone.0043760 22952759 10.1371/journal.pone.0043760 PMC3429500

[36] Bonnet E, Wuyts J, Rouzé P, Van de Peer Y. Evidence that microRNA precursors, unlike other non-coding RNAs, have lower folding free energies than random sequences. Bioinformatics 2004; 20: 2911–2917 doi: 10.1093/bioinformatics/bth374 15217813 10.1093/bioinformatics/bth374

[37] Zhang Q, Li J, Sang Y, Xing S, Wu Q, Liu X. Identification and Characterization of MicroRNAs in Ginkgo biloba var. epiphylla Mak. PLoS One 2015; 10: e0127184 doi: 10.1371/journal.pone.0127184 25978425 10.1371/journal.pone.0127184 PMC4433266

[38] Mi S, Cai T, Hu Y, Chen Y, Hodges E, Ni F, et al Sorting of small RNAs into Arabidopsis argonaute complexes is directed by the 5’ terminal nucleotide. Cell 2008; 133: 116–127 doi: 10.1016/j.cell.2008.02.034 18342361 10.1016/j.cell.2008.02.034 PMC2981139

[39] Delebinski CI, Twardziok M, Kleinsimon S, Hoff F, Mulsow K, Rolff J, et al A Natural Combination Extract of Viscum album L. Containing Both Triterpene Acids and Lectins Is Highly Effective against AML In Vivo. PLoS One 2015; 10: e0133892 doi: 10.1371/journal.pone.0133892 26244918 10.1371/journal.pone.0133892 PMC4526680

[40] Yang D, Du X, Liang X, Han R, Liang Z, Liu Y, et al Different Roles of the Mevalonate and Methylerythritol Phosphate Pathways in Cell Growth and Tanshinone Production of Salvia miltiorrhiza Hairy Roots. PLoS One 2012; 7: e46797 doi: 10.1371/journal.pone.0046797 23209548 10.1371/journal.pone.0046797 PMC3510226

[41] Kasahara H, Hanada A, Kuzuyama T, Takagi M, Kamiya Y, Yamaguchi S. Contribution of the mevalonate and methylerythritol phosphate pathways to the biosynthesis of gibberellins in Arabidopsis. J Biol Chem 2002; 277: 45188–45194 doi: 10.1074/jbc.M208659200 12228237 10.1074/jbc.M208659200

[42] Mulsow K, Enzlein T, Delebinski C, Jaeger S, Seifert G, Melzig MF. Impact of Mistletoe Triterpene Acids on the Uptake of Mistletoe Lectin by Cultured Tumor Cells. PLoS One 2016; 11: e0153825 doi: 10.1371/journal.pone.0153825 27088729 10.1371/journal.pone.0153825 PMC4835140

[43] Lee CH, Kim JK, Kim HY, Park SM, Lee SM. Immunomodulating effects of Korean mistletoe lectin in vitro and in vivo. Int Immunopharmacol 2009; 9: 1555–1561 doi: 10.1016/j.intimp.2009.09.011 19788934 10.1016/j.intimp.2009.09.011

[44] Franz H, Ziska P, Kindt A. Isolation and properties of three lectins from mistletoe (Viscum album L.). Biochem J 1981; 195: 481–484 7316963 10.1042/bj1950481 PMC1162912

[45] Eck J, Langer M, Möckel B, Baur A, Rothe M, Zinke H, et al Cloning of the mistletoe lectin gene and characterization of the recombinant A-chain. Eur J Biochem 1999; 264: 775–784 10491123 10.1046/j.1432-1327.1999.00638.x

[46] Voelter W, Wacker R, Franz M, Maier T, Stoeva S. Complete Structural Characterization of a Chitin-Binding Lectin from Mistletoe Extracts. J für Prakt Chemie 2000; 342: 812–818

[47] Ng TB, Chan YS, Ng CCW, Wong JH. Purification and Characterization of a Lectin from Green Split Peas (Pisum sativum). Appl Biochem Biotechnol 2015; 177: 1374–1385 doi: 10.1007/s12010-015-1821-x 26304129 10.1007/s12010-015-1821-x

[48] Schatler G, Urechl K, Grazi G, Giannattasio M. Viscotoxin Composition of the three European Subspecies of Viscum album. Lett Planta Med 1998; 64: 677–678 10.1055/s-2006-957553 17253311

[49] Sethupathy P, Megraw M, Hatzigeorgiou AG. A guide through present computational approaches for the identification of mammalian microRNA targets. Nat Methods 2006; 3: 881–886 doi: 10.1038/nmeth954 17060911 10.1038/nmeth954

[50] Kees UR. Gene expression signatures in lymphoid tumours. Immunol Cell Biol 2004; 82: 154–160 doi: 10.1046/j.0818-9641.2004.01236.x 15061768 10.1046/j.0818-9641.2004.01236.x

[51] Jiang P, Freedman ML, Liu JS, Liu XS. Inference of transcriptional regulation in cancers. Proc Natl Acad Sci U S A 2015; 112:7731–7736 doi: 10.1073/pnas.1424272112 26056275 10.1073/pnas.1424272112 PMC4485084

[52] Yang W, Ernst P. SET/MLL family proteins in hematopoiesis and leukemia. Int J Hematol 2017; 105: 7–16 doi: 10.1007/s12185-016-2118-8 27796741 10.1007/s12185-016-2118-8

[53] Goyama S, Schibler J, Cunningham L, Zhang Y, Rao Y, Nishimoto N, et al Transcription factor RUNX1 promotes survival of acute myeloid leukemia cells. J Clin Invest 2013; 123: 3876–3888 doi: 10.1172/JCI68557 23979164 10.1172/JCI68557 PMC3754260

[54] Oren M, Rotter V. Mutant p53 gain-of-function in cancer. Cold Spring Harb Perspect Biol 2010; 2: a001107 doi: 10.1101/cshperspect.a001107 20182618 10.1101/cshperspect.a001107 PMC2828285

[55] Hao Y, Yang J, Yin S, Zhang H, Fan Y, Sun C, et al The synergistic regulation of VEGF-mediated angiogenesis through miR-190 and target genes. RNA 2014; 20: 1328–1336 doi: 10.1261/rna.044651.114 24962367 10.1261/rna.044651.114 PMC4105756

[56] Sia D, Alsinet C, Newell P, Villanueva A. VEGF signaling in cancer treatment. Curr Pharm Des 2014; 20: 2834–2842 23944367 10.2174/13816128113199990590

[57] He Y, Ding Y, Liang B, Lin J, Kim TK, Yu H, et al A Systematic Study of Dysregulated MicroRNA in Type 2 Diabetes Mellitus. Int J Mol Sci 2017; 18: 456 10.3390/ijms18030456 PMC5372489 28264477

[58] Ozaki K, Awazu M, Tamiya M, Iwasaki Y, Harada A, Kugisaki S, et al Targeting the ERK signaling pathway as a potential treatment for insulin resistance and type 2 diabetes. Am J Physiol Endocrinol Metab 2016; 310: E643–E651 doi: 10.1152/ajpendo.00445.2015 26860984 10.1152/ajpendo.00445.2015

[59] Nagarajan A, Petersen MC, Nasiri AR, Butrico G, Fung A, Ruan HB, et al MARCH1 regulates insulin sensitivity by controlling cell surface insulin receptor levels. Nat Commun 2016; 7: 12639 doi: 10.1038/ncomms12639 27577745 10.1038/ncomms12639 PMC5013666

[60] Harada M, Luo X, Murohara T, Yang B, Dobrev D, Nattel S. MicroRNA Regulation and Cardiac Calcium Signaling. Circ Res 2014; 114: 689–705 doi: 10.1161/CIRCRESAHA.114.301798 24526675 10.1161/CIRCRESAHA.114.301798

[61] De Windt LJ, Lim HW, Bueno OF, Liang Q, Delling U, Braz JC, et al Targeted inhibition of calcineurin attenuates cardiac hypertrophy in vivo. Proc Natl Acad Sci U S A 2001; 98: 3322–3327 doi: 10.1073/pnas.031371998 11248077 10.1073/pnas.031371998 PMC30652

[62] Talukder MAH, Zweier JL, Periasamy M. Targeting calcium transport in ischaemic heart disease. Cardiovasc Res 2009; 84: 345–352 doi: 10.1093/cvr/cvp264 19640931 10.1093/cvr/cvp264 PMC2777954

[63] Murphy C. Iscador: Mistletoe in Cancer Therapy. New York: Lantern Books; 2001

[64] Hutchison ER, Okun E, Mattson MP. The therapeutic potential of microRNAs in nervous system damage, degeneration, and repair. Neuromolecular Med 2009; 11: 153–161 doi: 10.1007/s12017-009-8086-x 19763905 10.1007/s12017-009-8086-x PMC2757407

[65] Zhao Z, Zlokovic B V. Remote control of BBB: A tale of exosomes and microRNA. Cell Res 2017; 27: 849–850 doi: 10.1038/cr.2017.71 28674430 10.1038/cr.2017.71 PMC5518990

[66] Aryani A, Denecke B. Exosomes as a Nanodelivery System: a Key to the Future of Neuromedicine? Mol Neurobiol 2016; 53: 818–834 doi: 10.1007/s12035-014-9054-5 25502465 10.1007/s12035-014-9054-5 PMC4752585

[67] Xu B, Zhang Y, Du X-F, Li J, Zi H-X, Bu J-W, Yan Y, Han H, Du J-L. Neurons secrete miR-132-containing exosomes to regulate brain vascular integrity. Cell Res 2017; 27: 882–897 doi: 10.1038/cr.2017.62 28429770 10.1038/cr.2017.62 PMC5518987

[68] Albert SW. Mistletoe Man. New York: The Berkley Publishing Group; 2001

[69] Kawai T, Akira S. The roles of TLRs, RLRs and NLRs in pathogen recognition. Int Immunol 2009; 21: 317–337 doi: 10.1093/intimm/dxp017 19246554 10.1093/intimm/dxp017 PMC2721684

[70] van Rooij E. Introduction to the series on microRNAs in the cardiovascular system. Circ Res 2012; 110: 481–482 doi: 10.1161/CIRCRESAHA.111.257311 22302754 10.1161/CIRCRESAHA.111.257311

[71] Rothschild SI. microRNA therapies in cancer. Mol Cell Ther 2014; 2: 7 doi: 10.1186/2052-8426-2-7 26056576 10.1186/2052-8426-2-7 PMC4452061

[72] Trang P, Wiggins JF, Daige CL, Cho C, Omotola M, Brown D, et al Systemic delivery of tumor suppressor microRNA mimics using a neutral lipid emulsion inhibits lung tumors in mice. Mol Ther 2011; 19: 1116–1122 doi: 10.1038/mt.2011.48 21427705 10.1038/mt.2011.48 PMC3129804

[73] Cubillos-Ruiz JR, Baird JR, Tesone AJ, Rutkowski MR, Scarlett UK, Camposeco-Jacobs AL, et al Reprogramming tumor-associated dendritic cells in vivo using miRNA mimetics triggers protective immunity against ovarian cancer. Cancer Res 2012; 72: 1683–1693 doi: 10.1158/0008-5472.CAN-11-3160 22307839 10.1158/0008-5472.CAN-11-3160 PMC3319850

[74] Chin AR, Fong MY, Somlo G, Wu J, Swiderski P, Wu X, et al Cross-kingdom inhibition of breast cancer growth by plant miR159. Cell Res 2016; 26: 217–228 doi: 10.1038/cr.2016.13 26794868 10.1038/cr.2016.13 PMC4746606

[75] Mlotshwa S, Pruss GJ, MacArthur JL, Endres MW, Davis C, Hofseth LJ, et al A novel chemopreventive strategy based on therapeutic microRNAs produced in plants. Cell Res 2015; 521–524 doi: 10.1038/cr.2015.25 25721325 10.1038/cr.2015.25 PMC4387556

[76] Ng M, Yanofsky MF. Function and evolution of the plant MADS-box gene family. Nat Rev Genet 2001; 2: 186–195 doi: 10.1038/35056041 11256070 10.1038/35056041

[77] Phukan UJ, Jeena GS, Shukla RK. WRKY Transcription Factors: Molecular Regulation and Stress Responses in Plants. Front Plant Sci 2016; 7: 760 doi: 10.3389/fpls.2016.00760 27375634 10.3389/fpls.2016.00760 PMC4891567

[78] Lee HS, Kim YS, Kim SB, Choi BE, Woo BH, Lee KC. Isolation and characterization of biologically active lectin from Korean mistletoe, Viscum album var. Coloratum. Cell Mol Life Sci 1999; 55: 679–682 doi: 10.1007/s000180050324 10357236 10.1007/s000180050324 PMC11146893

[79] Schrader G, Apel K. Isolation and characterization of cDNAs encoding viscotoxins of mistletoe (Viscum album). Eur J Biochem 1991; 198: 549–553 1710983 10.1111/j.1432-1033.1991.tb16049.x

[80] Urech K, Schaller G, Jäggy C. Viscotoxins, Mistletoe Lectins and their Isoforms in Mistletoe (Viscum album L.) Extracts Iscador. Arzneimittelforschung 2011; 56: 428–434 10.1055/s-0031-1296808 16927522

[81] Pirrò S, Zanella L, Kenzo M, Montesano C, Minutolo A, Potestà M, et al MicroRNA from Moringa oleifera: Identification by High Throughput Sequencing and Their Potential Contribution to Plant Medicinal Value. PLoS One 2016; 11: e0149495 doi: 10.1371/journal.pone.0149495 26930203 10.1371/journal.pone.0149495 PMC4773123

